# Probabilistic risk assessment and water quality index of a tropical delta river

**DOI:** 10.7717/peerj.12487

**Published:** 2021-11-29

**Authors:** Osikemekha Anthony Anani, John Ovie Olomukoro

**Affiliations:** 1 Laboratory for Ecotoxicology and Forensic Biology, Department of Biological Science, Faculty of Science, Edo State University Uzairue, Auchi, Nigeria; 2 Department of Animal and Environmental Biology, Faculty of Science, University of Benin, Benin City, Edo State Nigeria

**Keywords:** Health risk, Water Quality Index, Carcinogenic risk factors, Heavy metals, Quality control.

## Abstract

Water plays a major role in supporting the wellness and life processes in living things as well as in the ecological structure’s stabilities. However, several environmental scientists have recounted the alarming menace unfit water quality portends as well as the shortfalls of its global utilization in various spheres of life. This study aims to determine the fitness of the Ossiomo River and its likely health risk impact when consumed or used for other domestic purposes. The outcome of the physicochemical and heavy metal characterization showed that most of the parameters surpassed the slated benchmarks. Findings from the study revealed a significant difference (*p* < 0.05) for water temperature, color, TDS, BOD_5_, HCO_3_, Na, Fe, Mn, and THC across the four stations respectively. Meanwhile, pH, salinity, turbidity, TSS, DO, Cl, P, NH_4_H, NO_2_, NO_3_, SO_4_, Zn, Cu, Cr, Ni, Pb, and V showed no significant (*p* > 0.05) across the four stations respectively. The pH level of the water was slightly acidic at the range of 4.40–6.82. The outcome of the computed water quality index showed that station 1 (66.38) was poor for human ingestion which was above the set slated benchmarks of 26–50. However, stations 2–4 (163.79, 161.79, and 129.95) were unsuitable for drinking which was above the set slated benchmarks of 100. The outcome of the health risk evaluation revealed that the hazard quotients (HQs) were considered greater than 1 (>1) for Cr (2.55). The hazard index (0.46) *via* the dermal pathway was <1 while the ingestion (4.35) pathway was >1. The sum of the HQs (4.81) was also > 1. Thus, there are possible non-carcinogenic health risks *via* direct ingestion of the water. The outcome from the carcinogenic risk for Pb, Cr, and Cd (6 × 10^–3^, 4.00 × 10^–1^, and 1.22 × 10^0^), was somewhat greater than the target goal (1.0 × 10^–6^ to 1.0 × 10^–4^) of carcinogenic risks stipulated by the United States Environmental Protection Agency for drinking water, respectively, especially for Cd. There might be a potential carcinogenic risk if the water is consumed when the metal contents are higher than the target limits set. Sustainable farming and treatment of wastes from industrial outputs should be the main management of this watercourse.

## Introduction

Surface or superficial water comprises water from reservoirs, lakes, ponds, springs, oceans, seas, and rivers. Though, such waters stemmed from dew, snow, and rainfall (precipitations). Most of these waters are used for various purposes such as industrial, agricultural, and domestic purposes globally (Manahan, 2010; Khan , Gani & Chakrapani, 2015; Shil, Singh & Mehta, 2019; Anani, Olomukoro & Enuneku, 2020; Anani, Olomukoro & Ezenwa, 2020). Surface water sourced from river watercourse has several intrinsic-physical and chemical properties that can sustain both plant and animal life forms. However, there are some environmental tendencies, several factors that can elevate and impact its background concentrations. These water bodies are often influenced by pollutants caused by natural and human activities (Kazi et al., 2009; Giridharan, Venugopal & Jayaprakash, 2010; Sener, Sener & Davraz, 2017; Anani & Olomukoro, 2018; Kumar, Singh & Ojha, 2018; Olomukoro & Anani, 2019). The degradation of the quality of water by these activities makes it unfit for defined purposes set for its usage.

Nonetheless, it has been recounted and estimated that over 1.1 billion of the populace of the world cannot assess potable and clean water; that is uninterrupted from pollution. More so, about four billion of the population of the world have been linked by exposure to different health-related diseases resulting in five million death globally (WHO, 2004; Azizullah et al., 2011).

Despite the major roles water play in supporting the wellness and life processes in living things as well as in the ecological structures stabilities, several environmental scientists have recounted the alarming menace unfit water quality portends as well as the shortfalls of its global utilization in various spheres of life (Okorafor et al., 2012; Casanovas-Massana & Blanch, 2013; Liang et al., 2013; Sojobi, Owamah & Dahunsi, 2014; Ayandiran et al., 2014; Dahunsi et al., 2014).

Human contact to heavy metals *via* different pathways (dermal and ingestion) in river water, is of utmost importance because of the associated problematic health severity it portends and likely food chain impacts. Previous research works have emphasized the health risk and water quality impact of surface, ground, and portable water globally (Cude, 2001; Song et al., 2012; Oboh & Agbala, 2017; Abbasnia et al., 2018a, 2018b; Ayandirana, Fawolea & Dahunsi, 2018; Enuneku et al., 2018; Emenike et al., 2019; Soleimani et al., 2018; Kamarehie et al., 2019; RadFard et al., 2019). Heavy metals (HMs) exposure and possible health risk impacts have been analyzed in various water bodies in Nigeria (Chinedu & Nwinyi, 2011; Kayode et al., 2011; Omole et al., 2015; Emenike et al., 2017).

So, there is an urgent need to forecast, evaluate, and address river water with possible pollutants that have a harmful influence on plants, animals and humans live to bring about sustainable management of our water resources.

Therefore, this study attempts to evaluate the probabilistic influence of heavy metals (HMs) in the surface water of Ossiomo River in the region of Ologbo, South-South Nigeria, to determine its consumption fitness and its likely health risk *via* oral and dermal pathways. However, several evaluations on the chemical and physical properties have been done on different parts of the River stretch. So far, no research work has been conducted on the quality of water and human health risk factors in this river which stands as a possible research gap.

## Materials & methods

### Study area

**Figure 1:**
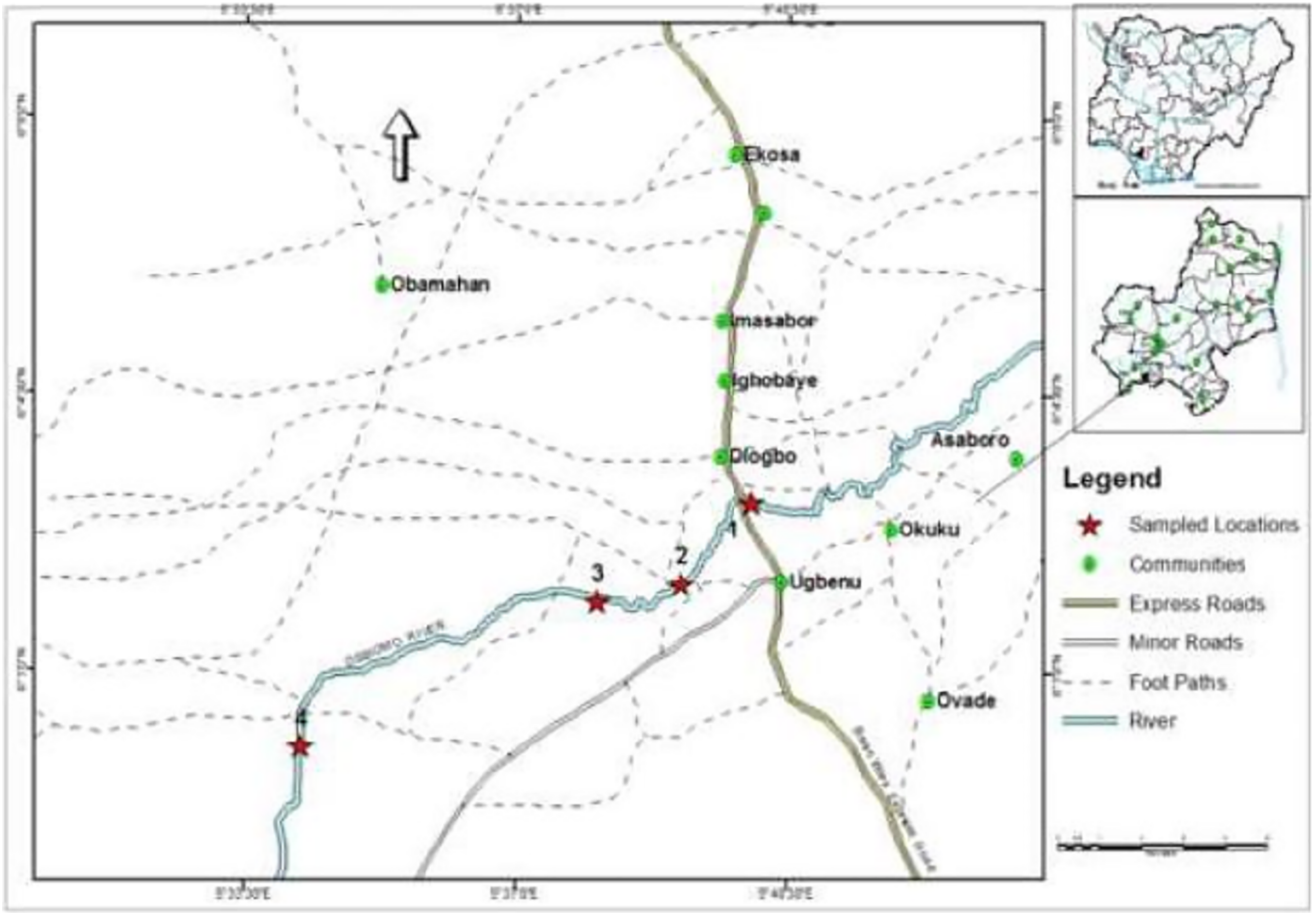
Map of Nigeria showing the study area (sub eco-communities) and sampling stations. GPS locations.

The study area Ossiomo River covers five sub-eco-communities which are Ekosa, Imasabor, Asaboro, Ovade, Ugbenu, and Okuku of geographical ranges: 6°03′.1″N (Latitude) to 5°40′.3″E (Longitude) Fig. 1. Two different sharply marked yearly seasons, wet and dry linked to these regions begins in early March and end in late November (wet season), and the dry season starts from November and ends in March. The mean precipitation for the sampling periods (2015 and 2016), fluctuated from 160.7–708.5 mm with the lowermost (158.4 mm), noted in the period of May 2015 and the topmost (708.5 mm), documented in the period of September 2015. The mean rainfall value within the sampling season was (434.6 mm).

The principal aquatic macrophytes here included; *Pandanus candelabrum*, *Elaeis guineensis*, *Azolla africana*, *Nymphaea lotus*, *Salvinia nymphellula*, *Echinochloa pyramidalis*, and *Pistia stratiotes*. Human activities within and around this river included; crude oil exploration, logging, fishing, boating, watercraft maintenance, and discharging of cassava wastes.

### Physical and chemical analysis

Samples were sourced from four labeled stations at periodic timing of 09.00 am and 12.00 pm on every one sampling day. Samples were collected for 18 months every two weeks every month. Each time, sampling began at station 1 and culminated at station 4. All samples were collected in reagent bottles and were ice chess at 4 °C in a large thermo cooler and taken to the laboratory for extraction and determination of several environmental concerned parameters (color, total suspended solids, total dissolved solids, biochemical dissolved oxygen, hydrogen carbonates, sodium, chlorine, potassium, ammonia nitrates, nitrites, nitrates, sulfates, iron, manganese, zinc, copper, chromium, cadmium, lead, nickel, vanadium, and total hydrocarbons) in consonance with acceptable standard methods (America Public Health Association (APHA), 1998).

### Field activities

The field water sampling involved the assessment of water temperature, DO (dissolved oxygen), TDS (total dissolved substances), pH, and EC (electrical conductivity) using a mercury-in-glass thermometer, Winkler A and B (Magnesium sulfate and Potassium iodide-Sodium Hydroxide), and Extech meter probes (Extsik ii) D 600 respectively. 1 mL of HNO_3_ was used to fix the heavy metal contents in the water collected in a clean 1-liter bottle. Similarly, a clean transparent 1-liter bottle was used to collect the THC (and total hydrocarbons) (Anani, Olomukoro & Ezenwa, 2020).

### Laboratory activities

Samples were taken to the laboratory in a thermo-cooler containing ice chests of temperature 4 °C for advanced analysis. The methods of the American Public Health Association (APHA) (2005) and Anani, Olomukoro & Ezenwa (2020) were used for the pretreatments, analytic measurements, and the determination of the following, color, turbidity, total suspended solids (TSS), chemical oxygen demand (COD), biochemical dissolved oxygen (BOD5), hydrogen carbonates, sodium, chlorine (Cl), potassium, ammonia nitrates, nitrites, nitrates, sulfates, iron, manganese, zinc, copper, chromium, cadmium, lead, nickel, vanadium, and total hydrocarbons. The instruments used were HACH UV/VIS Spectrophotometer model DR/2000, HACH Turbidimeter Model 2100p, and HACH Spectrophotometer at 890 nm Model DR 2000 for the measurement and determination of TSS, Turbidity, COD, phosphate, Na, hydrogen carbonates, and nitrate. The argento-metric technique was used to measure Cl, the turbidimetric technique was used to measure and determine sulfate. The Searchtech Dds-307 Benchtop digital electrical conductivity meter was used to determine the salinity in water. The metal contents were determined using the Atomic Absorption Spectrophotometer (AAS) Solaar 969 Unicam Series model.

The criteria for selecting the water quality parameters for the assessment is because over 85% of the population in this area depend solely on farming for their survival. As a result of this, various types of agricultural chemicals like herbicides, pesticides, and NPK fertilizers are employed in agricultural practices to improve farm products. In addition, agricultural and domestic wastes are poorly managed in this region. Contaminants like heavy metals, potassium, nitrogen, and phosphate from organic guano and fecal wastes have been assumed to reside in the soil and consequently washed *via* runoffs by rain or precipitation over time.

### Quality control

The worth of the diagnostic data was assured *via* the application of quality laboratory techniques and assurance like the examination of replicates, reagents blanks, the setting of standards, and operating methods. The samples collected from the field were analyzed in triplicates. For every triplicate, two standards *i.e*. 2.5 μg/L and one blank sample were analyzed correspondingly with an AAS (Atomic Absorption Spectrophotometer SOLAAR 969AA Unicam Series). After that, a recovery procedure was carried out in triplicate to ascertain the various metals. A mean recovery rate of 90.3 ± 0.75–96.7 ± 0.25% was established. Therefore, different calibration curves were improved by the use of QCSs (quality control standards) at each step of the sample evaluation. The chemicals used for the study were diagnostically procured and graded from Merck UK and Germany with a certified rate of purity of 99.89%. The glassware (Pyrex) used for this study was washed with ultra-deionized water and later plunged in HNO_3_ 10% overnight and rinsed later with ultra-deionized water. Lastly, they were dried in an oven at a temperature of 60 °C. The bottles (polyethylene) used were tightly covered before taking them for analysis (Chinedu & Nwinyi, 2011; Naveedullah Hashmi et al., 2013).

### Data analysis

Parametric analysis of variance (ANOVA) was used to compute the mean and standard deviation across the stations and the *p*-values were set at 0.05. Ramakrishna, Sadashivaiah & Ranganna (2009), Tyagi et al. (2013), Abbasnia et al. (2018a), (2018b), Soleimani et al. (2018), and RadFard et al. (2019) method of WQI (Water Quality Index) by the Weighted Arithmetic Index was employed to explain the range of quality of the water.

### Water Quality Index (WQI)

In this study, the Qi (quality rating scale) for individual parameters was estimated using the below equation:

$${\rm Qi\; } = \displaystyle{{{\rm \{ V\; actual} - {\rm V\; ideal)}} \over {\left( {V\; standard - V\; ideal} \right)}}*100$$

where Qi, V actual or actual value, and V ideal or ideal value equal to the quality evaluation of ith parameter for a sum of n WQ (water quality) parameters, the real value of the WQ parameter gotten from laboratory examination, and the perfect rate of that WQ parameter respectively, that can be sourced from a typical water quality table (Table 1).

**Table 1:** Relative weight, V standard, and V ideal of WQI parameters. The water parameters standards.

Number	Factor/parameters	WHO (2004) limit (V standard)	V ideal (Ramakrishna, Sadashivaiah & Ranganna, 2009; Tyagi et al., 2013; Abbasnia et al., 2018a, 2018b) protocol
1	Water temperature	35	0
2	pH	7.5	7
3	Colour	15	0
4	Turbidity	5	0
5	TSS	10	0
6	TDS	500	0
7	DO	7.5	14.6
8	BOD_5_	0	0
9	HCO_3_	200	0
10	Na	200	0
11	Cl	200	0
12	P	5	0
13	NH_4_H	1	0
14	NO_2_	1	0
15	NO_3_	10	0
16	SO_4_	500	0
17	Fe	1	0
18	Mn	0.05	0
19	Zn	1	0
20	Cu	0.1	0
21	Cr	0.05	0
22	Cd	0.01	0
23	Ni	0.05	0
24	Pb	0.05	0
25	V	0.01	0
26	THC	0.05	0

The pH of 7.0 and DO of 14.6 mg/L were used as standard V ideal values as documented and adopted by Ramakrishna, Sadashivaiah & Ranganna (2009), Tyagi et al. (2013), Abbasnia et al. (2018a), and (2018b) while the other parameters were equal to zero. However, the V standard or standard values are equal to the WHO (2004) standard limits for drinking water Table 1.

After estimating for the Qi, the Wi (weight) in the relative unit was estimated using the equation below:

$$Wi = 1/Si$$

where Wi, Si, and 1 stand for weight for the nth parameter, the allowable standard number for the nth parameter, and proportionality constant correspondingly.

Conclusively, the total WQI (water quality index) was estimated by totaling the Qi with the Wi linearly with the below equation:

$$WQI = \; \sum WiQi/\sum Wi$$

where Qi and Wi stand for quality rating and weight in relative units (Ramakrishna, Sadashivaiah & Ranganna, 2009; Tyagi et al., 2013; Abbasnia et al., 2018a, 2018b; Soleimani et al., 2018; RadFard et al., 2019) (Table 5).

**Table 5:** Summary of the health risk evaluation *via* dermal and ingestion pathways of metals in water samples sourced from Ossiomo River (Ologbo axis). Health risk.

Elements	Rfd ingestion (mg/kg/d)	Rfd dermal (mg/kg/d)	EXPing	EXP derm	HQ ing/derm	HQ ingestion	HQ dermal	∑HQS	∑HI ing/derm	CDI
Fe	0.7	1.4	0.036	0.00272	5,145.35	0.05	0.00	0.05	26.19	0.0362
Zn	0.3	0.06	0.003	0.00002	101,483,273.75	0.01	0.00	0.01	43.65	0.0033
Mn	0.014	0.06	0.014	0.00011	158,377.96	0.99	0.00	0.99	561.22	0.0141
Cu	0.4	0.0019	0.001	0.00001	13,499,099,610.00	0.00	0.01	0.01	0.62	0.0014
Pb	0.0035	0.0019	0.001	0.00002	93,764,937.00	0.23	0.01	0.24	17.77	0.0008
Cr	0.0003	0.00006	0.001	0.00001	557,632,930.35	2.55	0.20	2.75	13.10	0.0008
Cd	0.0005	0.00001	0.000	0.00000	116,389,254,373.90	0.47	0.18	0.66	2.62	0.0002
Ni	0.02	0.001	0.001	0.00006	456,285,178.92	0.04	0.06	0.10	0.65	0.0008
V	NS	NS	0.000	0.00001	ND	0.00	0.00	0.00	ND	0.0002
∑HI ing/derm						4.35	0.46	4.81		

**Note:**

ND means not detected and NS means not specified. Rfd (reference dosage), EXPing (exposure *via* ingestion contact), EXPderm (exposure *via* dermal contact), HQ ing/derm (hazard quotient of ingestion/dermal contacts), HQ ingestion (hazard quotient of ingestion contact), HQ dermal (hazard quotient of contact), ∑HQS (sum of hazard quotients), ∑HI (sum of hazard index), CDI (chronic daily intake), and ∑HI ing/derm (sum of hazard index of ingestion/dermal contacts).

### Health risk evaluation

#### Hazard quotient, hazard index, chronic daily intake, and carcinogenic risk

The health risk assessment for heavy metals in the surface water *via* dermal and ingestion routes were evaluated using the below equations:

$$E{X_{ping}} = \displaystyle{{Cwater \times IR \times EF \times ED} \over {BW \times AT}}$$

$$Ex{p_{derm}}\displaystyle{{Cwater \times {\rm SA} \times {\rm KP} \times {\rm ET} \times {\rm EF} \times {\rm ED} \times {\rm CF}} \over {{\rm BW} \times {\rm AT}}}$$

where *Exp*_*ing*_ means exposure dose *via* ingestion of water in mg/l/d and *Exp*_*derm*_ stands for exposure dose *via* dermal absorption in mg/l/d (US EPA, 1989; US EPA, 2004; Wu et al., 2009; Liang, Yang & Sun, 2011; Iqbal & Shah, 2012; Song et al., 2012; Fakhri et al., 2018a, 2018b; Qu et al., 2018). The assumptions used in the estimation of the dermal and ingestion pathways are as shown in Table 2.

**Table 2:** Assumptions or conventions use to quantify health risk exposure to heavy metals. Description of assumptions and conventions.

Exposure parameters	Units	Values
Levels of heavy metals in water (C_water_)	mg/l	–
Water ingestion rate (IR)	L/day	2.2
Exposure frequency (EF)	Days/year	360
Exposure duration (ED)	Year	30
Average body weight (BW)	Kg	70
Average time (AT)	Days	10,950
Exposed skin area (SA)	cm^2^	28,000
Exposure time (ET)	h/day	0.6
Unit conversion factor	L/cm^3^	0.001
Dermal permeability coefficient (Kp)	cm/h	0.0006
**Metals**	**Assumptions or coversions of metals used in this study**	
Zn	0.001	
Cu	0.001	
Mn	0.001	
Fe	0.001	
Cd	0.001	
Cr	0.001	
Pb	0.002	

**Note:**

Naveedullah Hashmi et al. (2013).

The equations for the estimation of the hazard quotient (HQ) and hazard index (HI) (non-carcinogenic risks) are as shown below:

$$H{Q_{\textstyle{{ing} \over {derm}}}} = \; \displaystyle{{\textstyle{{E{X_{ping}}} \over {Ex{p_{derm}}}}} \over {Rf{D_{\textstyle{{ing} \over {derm}}}}}}$$

$$H{I_{\textstyle{{ing} \over {derm}} = }} \mathop \sum \nolimits_{i = 0}^n H{Q_{\textstyle{{ing} \over {derm}}}}$$

where $H{Q_{\textstyle{{ing} \over {derm}}}}$ stands for hazard quotient *via* ingestion or dermal contact (unitless); and $Rf{D_{\textstyle{{ing} \over {derm}}}}$ refers to the oral/dermal reference dose (mg/kg/d) which was extracted from US EPA (1993), USEPA (2002), USEPA IRIS (2011), Iqbal & Shah (2012), Naveedullah Hashmi et al. (2013), and Anyanwu & Nwachukwu (2020) risk tables. *HI*_*ing/derm*_ stands for hazard index *via* ingestion or dermal contact (unitless). HI was introduced to appraise the sum probable for non-carcinogenic effects posed by additional pathways, which was the sum of the HQs (hazard quotients) from all applicable pathways. HI >1 and HQ > 1 displayed possibility for adversative influence on human health which might indicate concern for non-carcinogenic influence (Wu et al., 2009; Li & Zhang, 2010; Iqbal & Shah, 2012; Edokpayi et al., 2018; Fakhri et al., 2018a, 2018b; Qasemi et al., 2018; Shams et al., 2020).

The estimation of the possible CDI (chronic daily intake) of metals in the water was estimated using the equation below:

$$CDI = C*DI/BW$$

where C, DI, and BW indicated the levels of heavy metal in water (mg/L), the mean daily intake rate of 2.2 L/day, and the bodyweight of 70 kg corresponding as modified by Wu et al. (2009), Muhammad , Shah & Khan (2011), Dzulfakar et al. (2011), Edokpayi et al. (2018), Fakhri et al. (2018a), (2018b), Qasemi et al. (2018), and Shams et al. (2020).

For the carcinogenic risk pathway using ingestion, the equation for calculation is shown below:

$$Cring = EXping*Sfing\;$$

where Cr_ing_ means carcinogenic risk *via* ingestion, SF_ing_ means slope factor for carcinogenic risk *via* ingestion (mg/kg)-{(URF × 1,000 × URF (unit risk factor)}. To show the CR_ing_ values for Cd, Cr, and Pb, the SF_ing_ values for Cd, Cr, and Pb are 6.1E+03, 5.0E+02, and 8.5E+00, individually (De Miguel et al., 2007; Wu et al., 2009; Iqbal & Shah, 2012; Naveedullah Hashmi et al., 2013; Naz, Mishra & Gupta, 2016; Briki et al., 2017; Shams et al., 2020). The USEPA (2010) range (1.0E−06 to 1.0E−04) for carcinogenic risks were used to compare the valve gotten in this study.

## Results

### The physicochemical and heavy metal results of the Ossiomo River

The results of the physicochemical and heavy metals parameters are shown in Table 3 for stations 1–4 correspondingly. The study revealed a significant difference (*p* < 0.05) for water temperature, color, TDS, BOD_5_, HCO_3_, Na, Fe, Mn, and THC across the four stations respectively. Meanwhile, pH, salinity, turbidity, TSS, DO, Cl, P, NH_4_H, NO_2_, NO_3_, SO_4_, Zn, Cu, Cr, Ni, Pb, and V showed no significant (*p* > 0.05) across the four stations respectively.

**Table 3:** The summary of the physicochemical parameters of Ossiomo River used in the quantification of the WQI. Physicochemical parameters.

Parameters	Units	Station 1	Station 2	Station 3	Station 4	WHO (2004)	Significant values
		$\bar \times$ ± SD (Min-Max)	$\bar \times$ ± SD (Min-Max)	$\bar \times$ ± SD (Min-Max)	$\bar \times$ ± SD (Min-Max)		
Water Temperature	°C	26.19 ± 1.09	26.73 ± 0.87	26.99 ± 0.58	27.69 ± 0.58	NS	
		(26.60–28.10)	(24.90–28.00)	(26.10–28.00)	(24.4–29.10)		*p* < 0.05
pH		5.80 ± 0.56	5.48 ± 0.59	5.72 ± 0.52	5.64 ± 0.50	6–8	
		(4.94–6.82)	(4.11–6.12)	(4.84–6.50)	(4.70–6.24)		*p* > 0.05
Salinity	gl^−l^	0.05 ± 0.02	0.08 ± 0.02	0.08 ± 0.02	0.06 ± 0.02	NS	
		(0.03–0.08)	(0.05–0.13)	(0.05–0.11)	(0.03–0.09)		*p* < 0.05
Colour	Pt.Co	4.87 ± 2.40	6.66 ± 3.95	6.45 ± 3.49	5.38 ± 3.09	NS	
		(1.70–10.40)	(2.30–15.30)	(1.70–13.70)	(1.40–11.50)		*p* < 0.05
Turbidity	NTU	3.93 ± 2.14	5.54 ± 3.69	4.95 ± 2.65	4.29 ± 2.42	5	
		(1.20–8.40)	(1.80–13.90)	(1.10–10.50)	(0.90–7.80)		*p* > 0.05
TSS	mg l^−l^	6.15 ± 2.60	9.33 ± 4.45	8.48 ± 3.92	7.06 ± 3.17	NS	
		(2.80–12.50)	(4.70–19.40)	(2.80–16.30)	(2.10–14.00)		*p* > 0.05
TDS	mg l^−l^	60.28 ± 17.70	88.23 ± 23.30	82.10 ± 22.43	67.26 ± 17.09	1,000	
		(33.90–90.60)	(57.00–141.30)	(50.10–25.50)	(32.00–97.10)		*p* < 0.05
DO	mg l^−l^	6.23 ± 0.54	5.67 ± 0.69	5.67 ± 0.70	5.87 ± 0.38	NS	
		(5.40–7.10)	(4.80–6.90)	(4.10–6.70)	(5.20–6.40)		*p* > 0.05
BOD_5_	mg l^−l^	2.34 ± 0.57	3.44 ± 0.70	3.00 ± 0.82	2.44 ± 1.11	NS	
		(1.60–3.20)	(2.30–4.70)	(2.10–4.40)	(1.10–4.00)		*p* < 0.05
HCO_3_	mg l^−l^	20.78 ± 12.70	41.61 ± 11.93	39.50 ± 13.79	29.18 ± 15.13	NS	
		(12.20–54.20)	(24.40–61.00)	(24.40–59.20)	(6.10–54.90)		*p* < 0.05
Na	mg l^−l^	0.83 ± 0.42	1.12 ± 0.44	1.04 ± 0.45	0.93 ± 0.42	NS	
		(0.46–1.82)	(0.59–2.19)	(0.55–1.95)	(0.41–1.78)		*p* < 0.05
Cl	mg l^−l^	23.24 ± 18.78	43.31 ± 39.51	38.57 ± 34.94	26.88 ± 18.95	500	
		(7.00–73.20)	(15.20–150.30)	(11.50–26.90)	(10.70–82.80)		*p* > 0.05
P	mg l^−l^	0.65 ± 0.42	1.27 ± 1.06	1.26 ± 0.90	0.84 ± 0.59	NS	
		(0.12–1.30)	(0.33–3.28)	(0.35–3.17)	(0.16–1.95)		*p* > 0.05
NH_4_H	mg l^−l^	0.09 ± 0.05	0.20 ± 0.10	0.18 ± 0.16	0.12 ± 0.05	NS	
		(0.02–0.16)	(0.05-0.34)	(0.06-0.59)	(0.03–0.19)		*p* > 0.05
NO_2_	mg l^−l^	0.05 ± 0.03	0.14 ± 0.18	0.13 ± 0.19	0.08 ± 0.05	NS	
		(0.01–0.12)	(0.04–0.69)	(0.02–0.71)	(0.01–0.17)		*p* > 0.05
NO_3_	mg l^−l^	1.55 ± 0.59	2.96 ± 1.75	2.86 ± 1.64	1.77 ± 0.72	50	
		(0.74–2.48)	(0.93–6.27)	(0.77–5.10)	(1.11–3.19		*p* > 0.05
SO_4_	mg l^−l^	0.63 ± 0.35	1.07 ± 0.48	0.96 ± 0.40	0.82 ± 0.39	500	
		(0.27–1.49)	(0.53–2.30)	(0.47–1.84)	(0.21–1.71)		*p* > 0.05
Fe	mg l^−l^	0.68 ± 0.48	1.79 ± 1.22	1.50 ± 1.27	0.90 ± 0.50	0.4	
		(0.19–1.85)	(0.57–4.12)	(0.27–4.12)	(0.25–1.90)		*p* < 0.05
Mn	mg l^−l^	0.07 ± 0.05	0.16 ± 0.08	0.11 ± 0.07	0.09 ± 0.04	NS	
		(0.01–0.17)	(0.06–0.32)	(0.01–0.22)	(0.03–0.19)		*p* < 0.05
Zn	mg l^−l^	0.26 ± 0.16	0.67 ± 0.33	0.59 ± 0.36	0.39 ± 0.22	3	
		(0.09–0.55)	(0.24–1.35)	(0.09–1.29)	(0.11–0.81)		*p* > 0.05
Cu	mg l^−l^	0.03 ± 0.03	0.06 ± 0.04	0.06 ± 0.05	0.04 ± 0.03	0.05	
		(0.01–0.09)	(0.01–0.13)	(0.01–0.18)	(0.00–0.10)		*p* > 0.05
Cr	mg l^−l^	0.01 ± 0.01	0.04 ± 0.03	0.04 ± 0.05	0.02 ± 0.03	0.03	
		(0.00–0.05)	(0.00–0.13)	(0.00–0.18)	(0.00–0.09)		*p* > 0.05
Cd	mg l^−l^	0.01 ± 0.01	0.03 ± 0.02	0.03 ± 0.04	0.03 ± 0.02	0.01	
		(0.00–0.04)	(0.00–0.08)	(0.00–0.15)	(0.00–0.07)		*p* > 0.05
Ni	mg l^−l^	0.00 ± 0.00	0.01 ± 0.02	0.01 ± 0.02	0.00 ± 0.01	NS	
		(0.00–0.02)	(0.00–0.04)	(0.00–0.05)	(0.00–0.02)		*p* > 0.05
Pb	mg l^−l^	0.01 ± 0.02	0.04 ± 0.04	0.04 ± 0.04	0.01 ± 0.01	0.01	
		(0.00–0.08)	(0.00–0.12)	(0.00–0.17)	(0.00–0.04)		*p* > 0.05
V	mg l^−l^	0.00 ± 0.00	0.01 ± 0.01	0.01 ± 0.02	0.00 ± 0.00	NS	
		(0.00–0.01)	(0.00–0.03)	(0.00–0.05)	(0.00–0.01)		*p* > 0.05
THC	mg l^−l^	0.04 ± 0.03	0.11 ± 0.04	0.09 ± 0.06	0.07 ± 0.03	NS	
		(0.00–0.09)	(0.07–0.18)	(0.02–0.24)	(0.03–0.12)		*p* < 0.05

**Note:**

Unit of measurement: pH has no unit. *p* < 0.05 – Significant difference; *p* > 0.05 – No significant difference. NS: indicates not specified and N/A; indicates not available. WHO; World Health Organisation.

The minimum and maximum range of values obtained across the stations were: water temperature (24.40–29.10 °C), pH (4.40–6.82), colour (1.70–15.30 Pt.Co), turbidity (0.90–13.90 NTU), TSS (2.10–19.40 mg^–1^), TDS (2.10–19.40 mg^–1^), DO (4.10–7.10 mg^–1^), BOD_5_ (1.10–4.70 mg^–1^), Na (0.41–2.19 mg^–1^), Cl (7.00–15.30 mg^–1^), P (0.12–3.28 mg^–1^), NH_4_N (0.02–0.09 mg^–1^), NO_2_ (0.01–0.71 mg^–1^), NO_3_ (0.74–6.27 mg^–1^) and SO4 (0.21–2.30 mg^–1^), The ranks of the heavy metal concentrations in the water were in this rank: Fe > Zn > Mn > Cu > Cr > Pb > Cr > Ni > V.

### The results of the Water Quality Index in Ossiomo River

Table 4 shows the summary of the Water Quality Index (WQI) for the individual stations. The water quality index at stations 1, 2, 3, and 4 varied with minimum and maximum values of 3.38–197.24, 27.59–420.61, 18.68–728.50, and 15.09–311.6 respectively. The mean values of the WQI at stations 1, 2, 3, and 4 were 66.38 (12.73%), 163.79, 161.43, and 121.95 (87.27%) respectively.

**Table 4:** Summary of water quality index (WQI) for the individual stations in Ossiomo River (Ologbo axis) Benin city Nigeria. Water quality index.

	Station 1	Station 2	Station 3	Station 4
	Mean ± SD (Min-Max)	Mean ± SD (Min-Max)	Mean ± SD (Min-Max)	Mean ± SD (Min-Max)
WQI	66.38 ± 56.18 (3.38–197.2)	163.79 ± 106.51 (27.59–420.61)	161.43 ± 177.13 (18.68–728.50)	129.95 ± 72.86 (15.09–311.6)

**Note:**

Status of Water Quality Index (WQI) stating their descriptions: <50 (Excellent); 50–100 (Good); 100–200 (Poor); 250–300 (very poor) and > 300 (unsuitable for drinking) Ramakrishna, Sadashivaiah & Ranganna (2009), Abbasnia et al. (2018), and (2018b) and 0–25 (Excellent water quality) 26–50 (Good water quality) 51–75 (Poor water quality) 76–100 (Very poor water quality) and >100 (unsuitable for drinking) (Tyagi et al., 2013).

**Figure 2:**
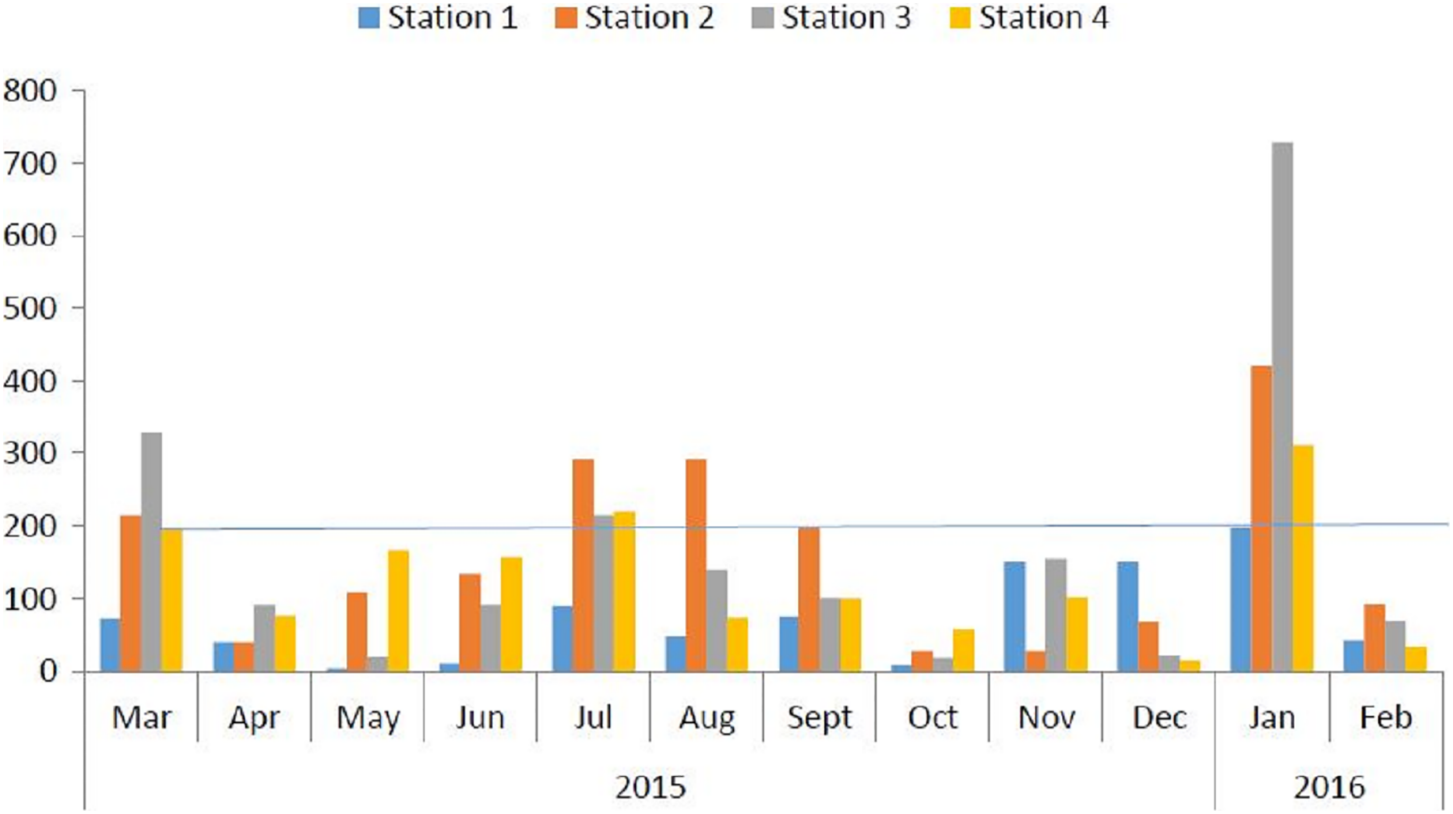
Monthly WQI across four stations in Ossiomo River. Data showing the monthly WQI of Ossiomo River.

Figure 2 shows the monthly variations of WQI across four stations in the Ossiomo River. The results showed that the month of January 2016, had the highest WQI.

### The results of the probabilistic health risk assessment of Ossiomo River

The results of the heavy metals exposure through dermal and ingestion routes of Ossiomo River were summarized in Table 5. The average ranks of exposure through ingestion (Exping) and exposure through dermal (Expderm) were observed in this order: Fe > Mn > Zn > Cu > Pb > Cr > Ni > Cd >V and Fe > Mn > Ni > Pb > Zn > V > Cr > Cu > Cd respectively (Table 5).

The result of the mean HQ of the metal was considered greater than 1 (>1) for Cr (2.55) (Table 5). The observed values for the HI *via* the ingestion (HIing) and dermal (HIderml) pathways were observed to be 4.35 and 0.46 respectively (Table 5). The sum of the HQs (4.81) was also > 1. The values obtained from the evaluation of the CDI for the selected heavy metals (Fe, Mn, Zn, Cu, Cr, Cd, Ni, Pb, and V) were 0.0362, 0.0033, 0.0141, 0.0014, 0.0008, 0.0008, 0.002, 0.0008, and 0.0002 respectively (Table 5).

The results of the CRing risk *via* ingestion for Pb, Cr, and Cd are shown in Table 6. The values obtained were 6 × 10^–3^, 4.00 × 10^–1^, and 1.22 × 10^0^ respectively.

**Table 6:** Summary of cancer risk (cr) assessment for some selected metals in water samples from Ossiomo River (ologbo axis) through dermal and ingestion pathways during the sampling periods. Cancer risks.

Elements	EXP_ing_	Sf_ing_	CR
Pb	0.001	8.50E+00	6.80E−03
Cr	0.001	5.00E+02	4.00E−01
Cd	0.000	6.10E+03	1.22E+00

**Note:**

EXPing, exposure vía ingestión pathway; Sfing, slope factor of the ingestión pathway; CR, cáncer risk.

## Discussion

In this study, the physicochemical and heavy metal assessment carried on Ossiomo River showed that some parameters were slightly higher than the WHO (2004, 2008) standard limits. The pH level of the water was slightly acidic. The variations in the concentrations of the water parameters may be a result of seasonality. This finding is closely related to what was obtained in previous studies by Oboh & Agbala (2017) in Siluko River southern Nigeria, Ayandirana, Fawolea & Dahunsi (2018) in Oluwa River Southwestern Nigeria, and Emenike et al. (2019) to similar water bodies in South-south Nigeria which have the same environmental factors influencing the water characteristics.

On the other hand, when the water parameters were compared with the WHO standards for drinking water, the findings of this study revealed ecological parameters like water temperature, turbidity, dissolved oxygen, biological dissolved oxygen, phosphate, iron, manganese, nickel, and lead which were lesser than the WHO (2004, 2008) standard limits. The contents of the physicochemical and heavy metal record in this river ecosystem were observed to be a function of anthropogenic activities located close to the river (Anani & Olomukoro, 2018; Kumar, Singh & Ojha, 2018; Olomukoro & Anani, 2019; Olatunji & Anani, 2020).

This study showed that the quality of water at station 1 was poor for human consumption. Station 1 had a value that was more than the benchmark of 26–50 for good water as established by Tyagi et al. (2013). Stations 2–4 were considered unsuitable for drinking with values that were more than the benchmark of 100 for both excellent and good water, as established by Ramakrishna, Sadashivaiah & Ranganna (2009). The finding was different from what was obtained by Oboh & Agbala (2017) in the range of 11.24–16.15 in Siluko River Southern Nigeria. However, a similar finding was reported by Akinbile & Omoniyi (2018) with WQI of 44.61 and 44.91 at River Ogbese, Nigeria when classified and interpreted according to the methods of Pradyusa et al. (2009) and Elizabeta et al. (2010) respectively. The WQI of 259.04 and 236.51 were reported by Iwar, Utsev & Hassan (2021) for River Benue Nigeria. The authors classified the water as poor and unfit for drinking purposes. Etim et al. (2013), reported the WQI of 55.05–84.94 for different water streams in Niger Delta water in Nigeria which were considered poor for drinking purposes. Similarly, Ogbozige et al. (2017) reported the WQI of 44.95–60.80 from River Kaduna, Nigeria. Edwin & Murtala (2013), Ochuko et al. (2014), Otene & Nnadi (2019), and Madilonga et al. (2021) reported the WQI of 41.3–52.9, 51–70, 29.732–79.342, and WQI > 100 for River Asa Ilorin, Nigeria, River Ase Southern Nigeria, Minichinda Stream, Port Harcourt, Nigeria, and Mutangwi River, Limpopo Province, South Africa respectively. The water was classified as poor for human consumption.

In a relative study done by Ramakrishna, Sadashivaiah & Ranganna (2009) in Tumkur Taluk India, the authors reported the WQI values of 89.21 to 660.56 which was about 63% of the water, was considered poor and 27% was considered okay for drinking. Abbasnia et al. (2018a), and (2018b) investigated the surface water in Baluchistan province in Iran. The authors reported that about 25% of the water was evaluated poor for consumption, 25% was excellent, and 50% was okay for drinking. RadFard et al. (2019) investigated the WQI of groundwater in Bardaskan villages Iran of 23.3 and 13.3% poor and very poor correspondingly. Meanwhile, 3.3 and 60% of the water were excellent and good respectively.

It was observed that the quality of water in this ecosystem was likely influenced by both anthropogenic; mainly agronomic activities, petrochemical influences, and natural processes. This finding is similar to the work by Naveedullah Hashmi et al. (2014) on the evaluation of Siling surface reservoir in China which linked human activities as one of the major sources of water contamination. This was also collaborated by the woks of Anani & Olomukoro (2018), Kumar, Singh & Ojha (2018), Olomukoro & Anani (2019), and Olatunji & Anani (2020). More so, the findings from the WQI in this study revealed that the water was influenced by seasonality and Cd sourced from agronomic influence. This leads to a change in the water quality characteristics and possible health risks if the water is consumed without proper treatment.

The potential health risk from heavy metals exposure through the dermal and ingestion routes of the water sourced from Ossiomo River after quantification and evaluation was considered not too high in terms of possible human impacts. This finding is not far different from what was obtained by Naveedullah Hashmi et al. (2014); 41.0 μg/L for Fe, Mn 37.32 μg/L, and Cd 1.18 μg/L from Siling surface reservoir in China for the summer/raining season period.

The observed values for the HQ and HI *via* the ingestion (HIing) pathway were considered to be greater than 1 (>1). Thus, there were possible non-carcinogenic health risks *via* direct ingestion of the water. Similar results were also obtained by Li & Zhang (2010) for Han River, China. On the contrary, Naveedullah Hashmi et al. (2014) reported the HQ (0.554) and HI (0.985) < 1 for the ingestion and dermal pathways. On the other hand, Anyanwu & Nwachukwu (2020) evaluated the possible ingestion hazard a South-eastern Nigeria River might pose if consumed without treatment. In their study, an HI >1 for all the stations was recorded. This was dissimilar from what was obtained in this study. However, an HQ >1 was obtained by the same authors for Fe, Cd, and Mn. Contrarily in this study, an HQ > 1 was obtained for only Cr.

It was obvious that in the ingestion pathway, the observed values fluctuated within the safe unity limit of <1 for the HQ and > I for the HI. These findings indicated non-carcinogenic health risks *via* direct ingestion contact with inhabitants. This is similar to what was obtained by Iqbal & Shah (2012) on the hazard quotients (HQ >1) of heavy metals in Simly (23.00) and Khanpur (18.85) freshwater lakes Pakistan respectively. There was no potential risk posed by the dermal pathway. However, most of the ∑HIing/derm of metals which were Fe, Zn, Mn, Cu, Cr, and Ni, fluctuated within the unity limit set by US EPA (2004). The likely main contributors to the non-carcinogenic health risks in this current study could be linked to Cr and Mn influence on the ecosystem. This finding is not far different from the works of Naveedullah Hashmi et al. (2013, 2014). He et al. (2004) and Wu et al. (2009) proposed that insecticides, from farm practice and sewage from domestic activities, might increase the concentration of Zn, Fe, and Mn. This, in turn, can affect the water quality parameters. This shows that the heavy metals present in the ecosystem may harm human health if consumed without proper treatment using conventional methods like boiling and chlorination.

The results of the CRing risk *via* ingestion for Pb, Cr, and Cd were slightly higher than the target remedial goal of carcinogenic risks (1.0 × 10^–6^ to 1.0 × 10^–4^) for surface water intake as set by (US EPA, 1989; US EPA, 2004; Vieira et al., 2011; Yu, Fang & Ru, 2010). This finding was quite dissimilar to what was obtained by Iqbal & Shah (2012) in Simly and Khanpur lakes for Pb (5.4 × 10^1^ and 5.9 × 10^1^), Cr (1.2 × 10^3^ and 7.2 × 10^2^), and Cd (3.2 × 10^3^ and 3.9 × 10^3^), respectively. George, David & Joseph (2015), obtained 1.50 × 10^–6^ and 50.15 × 10^–7^ for Cr and Cd respectively. The implication here is that there might be a potential carcinogenic risk if the water is consumed when the metal contents are higher than the target limits set.

## Conclusions

The computed details of all the values of the chemical elements, WQI, and health indices gave a better picture of the overall status of Ossiomo River and also reflect the parameters of most importance. The WQI indicated that likely, station 1 is fit for consumption as at the time of this study and indicated stations 2, 3, and 4 as unfit for consumption. The health risk assessment revealed likely non-carcinogenic risks *via* the ingestion contacts and possible carcinogenic risks if the water is consumed when the metal contents are higher than the target limits set. Sustainable farming and treatment of wastes from industrial outputs should be the main management of this watercourse. Proper treatment using conventional methods like boiling and chlorination should be recommended.

## Supplemental Information

10.7717/peerj.12487/supp-1 Supplemental Information 1 Water Quality Index data.

## Additional Information and Declarations

### Data Availability

The following information was supplied regarding data availability: The raw data is available in the Supplemental File.
